# Hook2 contributes to aggresome formation

**DOI:** 10.1186/1471-2121-8-19

**Published:** 2007-05-31

**Authors:** Györgyi Szebenyi, W Christian Wigley, Branden Hall, Aaron Didier, Michelle Yu, Philip Thomas, Helmut Krämer

**Affiliations:** 1 Center for Basic Neuroscience, University of Texas, Southwestern Medical Center, 6000 Harry Hines Blvd., Dallas, Texas 75390-9111, USA; 2 Department of Physiology, University of Texas, Southwestern Medical Center, 6000 Harry Hines Blvd., Dallas, Texas 75390-9040, USA; 3 Reata Pharmaceuticals, Inc., 2801 Gateway Drive, Irving TX 75063-2648, USA

## Abstract

**Background:**

Aggresomes are pericentrosomal accumulations of misfolded proteins, chaperones and proteasomes. Their positioning near the centrosome, like that of other organelles, requires active, microtubule-dependent transport. Linker proteins that can associate with the motor protein dynein, organelles, and microtubules are thought to contribute to the active maintenance of the juxtanuclear localization of many membrane bound organelles and aggresomes. Hook proteins have been proposed to serve as adaptors for the association of cargos with dynein for transport on microtubules. Hook2 was shown to localize to the centrosome, bind centriolin, and contribute to centrosomal function.

**Results:**

Here we show that overexpression of hook2 promotes the accumulation of the cystic fibrosis transmembrane regulator in aggresomes without altering its biochemical properties or its steady state level. A dominant negatively acting form of hook2 that lacks the centriolin binding C-terminal inhibits aggresome formation.

**Conclusion:**

We propose that hook2 contributes to the establishment and maintenance of the pericentrosomal localization of aggresomes by promoting the microtubule-based delivery of protein aggregates to pericentriolar aggresomes.

## Background

Intracellular protein aggregates form when misfolded proteins accumulate in cells because of malfunctioning or overloading of either the quality control pathways that recognize and route defective proteins for degradation or the elements of the actual degradative pathway [1]. Aggregates distribute randomly throughout the cell and can be associated with pathogenic changes; for example, they may block microtubule-based transport [2] or sequester components of the ubiquitin-proteasome system [3].

One of the cellular responses to potentially pathogenic aggregates of misfolded proteins is their dynein-mediated retrograde transport along microtubules to the centrosome [4,10] where they are enriched together with elements of the protein-folding and degradation machinery in pericentriolar structures, called aggresomes [4-9].

Aggresomes may protect cells by sequestering harmful protein aggregates and enhancing their degradation either by concentrating them together with proteasome subunits or by triggering their uptake into autophagosomes and delivery to lysosomes [10]. Experimentally induced aggresomes stirred general interest, because they are similar in composition and morphology to inclusion bodies found in brains of patients who died of neurodegenerative diseases [6,7,9,11].

The most studied protein that accumulates in aggresomes is the cystic fibrosis transmembrane regulator (CFTR). Similar to some other integral membrane proteins that have large hydrophobic regions [12], over-expressed CFTR is inefficiently processed [13]. This is even more pronounced for a prevalent mutation in cystic fibrosis patients, the ΔF508-CFTR deletion mutant, which is degraded by the proteasome [14,15]. When the degradation of CFTR is inhibited, CFTR accumulates in pericentrosomal aggresomes [7,9]. The retrograde transport of CFTR and other misfolded proteins depends on the integrity of the microtubule cytoskeleton and the association of dynein with the cargo-binding dynactin complex [4,16,17].

Linker proteins that associate with dynein, organelles, and microtubules facilitate the loading of cargos for retrograde transport and contribute to the establishment and active maintenance of the juxtanuclear localization of organelles [18], and thus they may play a role in the formation of aggresomes. Hook-related proteins were proposed to function as linker proteins [19]. Hook proteins are composed of a conserved N-terminal domain, a central coiled-coil, and a more divergent C-terminal domain that has been implicated in the binding of each of the hook proteins to a different class of organelles [20,21].

Data from several studies suggested that hook proteins may modulate microtubule based transport. The first identified member of the hook family, *Drosophila hook*, was originally discovered based on a defect in endocytic trafficking [22,23]. The characterization of a *C. elegans *homolog, zyg-2, revealed a function in binding and linking centrosomes to nuclei through the microtubule cytoskeleton [24]. We recently found that mammalian hook2 also localizes to centrosomes, in this case through a direct interaction with centriolin [21]. In addition, altering hook2 levels or function led to the accumulation of both endogenous and overexpressed proteins at the centrosome, raising the possibility that hook2 may influence aggresome formation.

Here, we are using mutant CFTR, which is well-known to accumulate in aggresomes [7,9], to examine whether hook2 contributed to the accumulation of misfolded proteins around the centrosome. We found that altering hook2 activity by overexpressing hook2 or using dominant-negative hook2 proteins changed the distribution of aggresome constituents. We have considered the possibility that hook2 induced changes in the activities of the protein degradation pathway, such as ubiquitination, however we found no evidence for hook2-induced biochemical changes in CFTR. Therefore, we propose a model wherein hook2 influences aggresome formation by interfering with the functioning of the centrosome in the coordination of vectorial intracellular transport.

## Results

### Hook2 co-localizes with aggresomes at the centrosome

**Figure 1:**
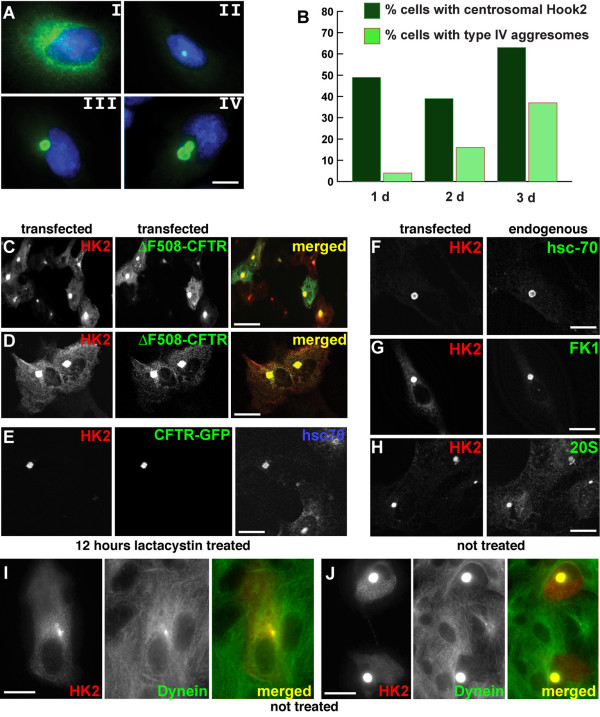
**Hook2 co-localizes with aggresomes at the centrosome and promotes aggresome formation**. Vero cells were transfected to express Hook2. (A) Immunostaining revealed Hook2 either diffusely distributed (type I) or enriched at centrosomes (types II-IV) and [21]. Type IV refers to large aggresomes (> 4 μm). (B) Cells containing distinct Hook2 patterns were counted after the indicated days of expression. The fraction of cells with some hook2 at centrosomes (type II-IV combined) only modestly increased from 1 to 3 days of expression. The fraction of cells in which co-expressed CFTR accumulated in large juxtanuclear aggresomes (type IV) increased about ten-fold from day 1 to 3. (C-D) Cells were transfected to express hook2 and ΔF508-CFTR or (E) hook2 and CFTR-GFP. After 24 hours, 10 μM lactacystin was added and cells were incubated for an additional 16 hours before either being double-stained (C-D) for hook2 (red in merged images) and co-expressed CFTR (green) or triple stained (E) for hook2, CFTR and endogenous hsc70. (F-J) Hook2 was expressed in the absence of CFTR and lactacystin and tested for co-localization with the endogenous aggresome markers hsc-70, ubiquitin (FK1), the 20S proteasome subunit or cytoplasmic dynein, as indicated. Merged images in (I and J) indicate the accumulation of dynein in hook2-induced type II (I) and type IV (J) aggresomes.

Endogenous and overexpressed hook2 localizes to the centrosome, as we previously showed by colocalization with the centrosomal markers ninein and gamma-tubulin, at the center of the radial microtubule array [21]. The juxtanuclear localization of over-expressed hook2-constructs resembled the centrosomal distribution of endogenous hook2 in an accentuated form [Fig. 1A and ref. [21]]. Centrosomal accumulation of hook2 gradually increased upon continued expression over 3 days with only a modest change in the number of cells with centrosomally localized hook2 (Fig. 1B). We have previously shown that centrosomal accumulation of hook2 did not disrupt the microtubule network or the Golgi complex [21], indicating that hook2 overexpression did not disrupt the structural integrity of cells.

Because overexpressed hook2 accumulated around the centrosome, we wondered whether hook2 and components of aggresomes co-localized. To induce aggresomes experimentally, the proteasome inhibitor lactacystin [26] was applied to cells that co-expressed ΔF508-CFTR and hook2. After 12 h of lactacystin treatment, aggregates of CFTR co-localized with hook2 in juxtanuclear aggresomes (Fig. 1C–E).

### Over-expressed hook2 promotes the juxtanuclear accumulation of aggresome components

Hook2 not only co-localized with lactacystin-induced and CFTR-containing aggresomes, but also induced aggresome formation in the absence of proteasome inhibitors and CFTR, as indicated by the juxtanuclear accumulation of endogenous hsc70, ubiquitin, and the 20S component of the proteasome in hook2 transfected cells (Fig. 1F–H).

Dynein-mediated transport of cargo to aggresomes is necessary for their formation, but in this process the dynein motor also becomes concentrated within aggresomes [4,16]. Consistent with these results, we observed that aggresomes induced by hook2 were enriched in dynein, even in the absence of lactacystin. This accumulation was visible already at the earliest stages in hook2-induced aggresome formation when little hook2 had accumulated (Fig 1I) and became more pronounced as the amount of centrosomal hook2 accumulation increased (Fig 1J).

**Figure 2:**
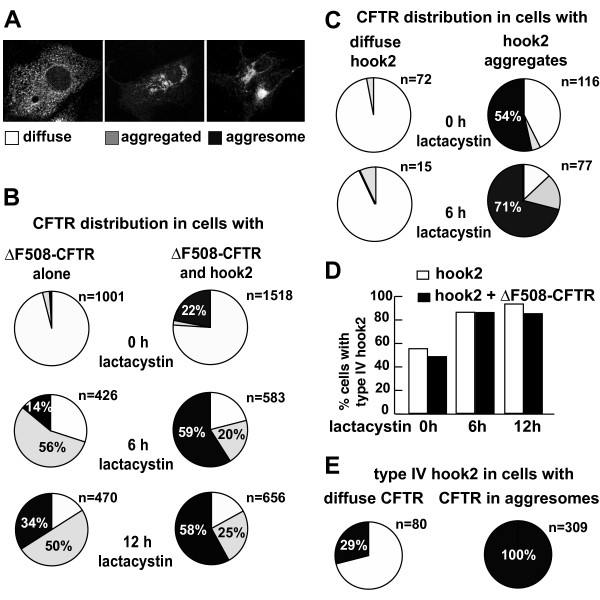
**Effects of hook2 on aggresomes formation**. (A) Vero cells were transfected to express CFTR. Immunofluorescence staining revealed CFTR either diffusely distributed □, aggregated , or accumulated in aggresomes ■. (B-C) Cells were transfected to express ΔF508-CFTR with or without hook2. After 24 h 10 μM lactacystine was added for the indicated times, before cells were stained for CFTR and the fractions of cells exhibiting distinct patterns were counted. Pie charts show an increase in the percentage of cells with aggresomal CFTR in cells that co-express hook2, compared to cells that express ΔF508-CFTR alone. Even though lactacystin treatment increased the incidence of ΔF508-CFTR in aggresomes, hook2 promoted aggresome formation in the absence of lactacystin (0 h). (C) The aggresome-promoting effect of hook2 was even more pronounced when cells in which hook2 was enriched at centrosomes were considered separately from those in which hook2 was distributed diffusely. (D-E) Cells were transfected to express hook2 alone or with ΔF508-CFTR and the fraction of cells with type IV hook2 distribution (See Fig. 1A) was counted. (D) In contrast to the effect of hook2 on CFTR (shown in B and C), the presence of CFTR did not change the percentage of cells with type IV hook2. 6 or 12 hours of lactacystin exposure increased the percentage of cells with hook2 aggresomes, but CFTR had no additional effect. (E) CFTR distribution strongly correlated with that of hook2 since in 100% of cells with aggresomal CFTR hook2 was co-enriched at centrosomes.

Moderately overexpressed CFTR is found throughout the secretory system including the ER and on the plasma membrane (Fig. 2A "diffuse"). With increased expression or upon addition of proteasome inhibitors, misfolded CFTR forms multiple aggregates that tend to localize close to the nucleus but are found throughout the cell (Fig. 2A "aggregated") and over time concentrates in compact juxtanuclear aggresomes (Fig. 2A "aggresome"). Time lapse imaging has previously captured these three types of distributions as stages along the pathway of aggresome formation [4]. After 6 hrs of lactacystin treatment, CFTR is found in aggregates in 70% of cells, but only in 14% of these it has accumulated yet in a single prominent aggresome (Fig. 2B). While the percentage of cells with aggregates modestly increased to 84% after 12hrs, the incidence of aggresomes more than doubled to 34%.

Co-expression with hook2 caused a dramatic increase in the frequency of ΔF508-CFTR aggresomes at each time point. The hook2-induced increase of CFTR in aggresomes was most obvious without treatment with lactacystin (from 2% to 22%, n > 1000; Fig. 2B, 0 h). Hook2 coexpression did not result in an increase in the percentage of cells with aggregated CFTR after 6 or 12 hrs in the presence of lactacystin. However, at every time point, the frequency of aggresomes at least doubled upon hook2 coexpression.

The effect of hook2 on the induction of aggresomes without lactacystin treatment was even more pronounced when the distribution of ΔF508-CFTR was considered separately in cells with hook2 accumulation at the centrosome and in cells with diffuse hook2: 54% of cells with centrosomal hook2 had ΔF508-CFTR-positive aggresomes compared to only 3% of cells with a diffuse hook2 distribution (Fig. 2C). Therefore, centrosomally localized hook2 promotes aggresome formation.

We also examined the effect of CFTR co-expression on the distribution of hook2. If the effect of hook2 on CFTR accumulation in aggresomes is only due to an unspecific increase in the level of misfolded proteins, then misfolded CFTR should have a reciprocal effect on hook2. However, in contrast to the dramatic effect of hook2 on ΔF508-CFTR localization, ΔF508-CFTR had no effect on hook2 localization. Hook2 juxtanuclear aggregates were seen in the same percentage of cells with and without ΔF508-CFTR (Fig. 2D). Inhibition of proteasome activity increased the number of cells with juxtanuclear hook2 accumulation, but ΔF508-CFTR had no effect on hook2 distribution. Moreover, 100% of ΔF508-CFTR aggresomes were also enriched in hook2. By contrast, only 29% of the cells with diffusely distributed ΔF508-CFTR contained hook2-positive type IV aggresomes (Fig. 2E). The finding that hook2 distribution at the centrosome is not influenced by the level of misfolded and aggregated ΔF508-CFTR argues against hook2 simply accumulating in aggresomes as misfolded proteins. Taken together, these date indicate that centrosomally located hook2 promotes aggresome formation.

### Interference with hook2 function alters CFTR aggresome formation

To further test a possible role for hook2 in aggresome formation, we attempted different approaches to interfere with hook2 function. Because so far we have not been successful in significantly knocking down hook2 levels by RNAi, we expressed hook2 truncations that are predicted to act as dominant negatives. This approach has previously been successful in *Drosophila *where phenotypes very similar to those of the *hook*^11 ^null allele resulted from the expression of dHook proteins that were truncated at their N- or C-termini but retained the coiled-coil dimerization domain [22].

**Figure 3:**
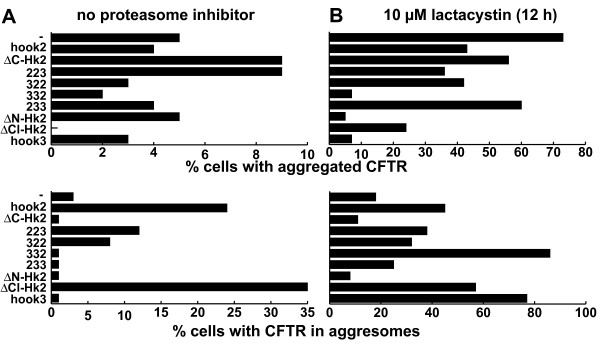
**Effect of hook proteins on CFTR distribution**. Hook proteins and CFTR were co-expressed in Vero cells for 36 hours in the absence (A) or presence (B) of 10 μM lactacystin for the final 12 hours. Graphs show the fraction of cells with aggregated CFTR (top) or with CFTR in juxtanuclear aggresomes (bottom), as shown in Figure 2A. In the rest of cells, CFTR was distributed diffusely throughout the cell. At least 400 cells were counted for each construct in at least two separate assays. For hook2 and hook2 truncations more than 5000 cells from at least ten separate assays were counted and yielded similar results. Results shown are from one representative experiment in which all constructs were used in parallel. While absolute numbers changed significantly in different experiments, the relative effect of different constructs was highly consistent between different experiments.

Thus, we tested whether corresponding hook2 truncation mutants altered aggresome formation. We counted the number of cells with distinct CFTR distributions in Vero cells (Figure 3A) or HEK293 cells (data not shown) that expressed ΔF508-CFTR for 48 hours together with various hook2 constructs. Especially ΔC-hook2, which lacks the centriolin binding site of hook2 [21], but retains its dimerization domain, was expected to interfere with the function of the endogenous hook2 protein.

In the absence of lactacystin, CFTR distribution was mildly altered by co-expression of ΔC-hook2: it reduced the fraction of cells with CFTR aggresomes to 1% from 3% observed with CFTR alone, and increased the fraction of cells with aggregated CFTR from 5% to 9%. By contrast, under these conditions co-expression of full-length hook2 increased the fraction of cells with CFTR in aggresomes from 3% to 24%. This effect is specific, as co-expression of hook3 or the hook chimeras 332 and 233, which contain mostly hook3 sequences, did not increase CFTR aggregation.

The presence of lactacystin (Figure 3B) caused a large increase in the percentage of cells with CFTR aggregates and aggresomes [7,9]. However, even under these conditions hook2 proteins retained their effect on aggresome formation. Compared to CFTR-only transfected cells, cotransfection with wild-type hook2 doubled the fraction of cells with aggresomes and made them as frequent as cells with aggregates. By contrast, the presence of the dominant negative ΔC-hook2 caused a reduction in the percentage of cells with perinuclear aggregates and aggresomes consistent with hook2 playing a role in their formation.

N-terminally truncated hook2 had an effect similar to that of ΔC-hook2. In the presence of lactacystin, co-expression of ΔN-hook2 reduced by ~50% the percentage of cells with ΔF508-CFTR in aggresomes compared to CFTR-only transfected cells. Similar results were obtained in five separate experiments in VERO cells (Figure 3B) as well as HEK293 cells (data not shown). The interpretation of these results is complicated by our previous finding that ΔN-hook2 can recruit centrosomal proteins such as ninein and thus may indirectly interfere with retrograde transport [21]. Importantly, no inhibitory effect on aggresome formation was observed upon co-expression of hook3 (Fig. 3B). On the contrary, hook3 over-expression enhanced the effect of lactacystin and caused a further increase in aggresomes; this is likely due to an unspecific effect, since Hook3 itself accumulates at centrosomes when proteasome activity is inhibited (data not shown). Consistent with this notion, co-expression of C-terminally truncated hook3 or hook1 did not result in a reduction in aggresomes (data not shown), further supporting a specific function for hook2 in aggresome formation.

In an attempt to pinpoint a specific domain in hook2 responsible for its effect on aggresome formation, we tested several chimeras between hook2 and hook3. We found that chimeras of hook2 and the C- or the N-terminus of hook3 enhanced aggresome formation in the absence of lactacystin (Fig. 3A). Interestingly, this parallels our previous findings with these chimeras showing that domains partially redundant between hook2 and hook3 contribute to centrosomal localization of hook2 [21].

### Hook2 changes CFTR distribution without an obvious biochemical change

Expression of hook2 could affect aggresome formation by altering biochemical properties of CFTR that cause it to aggregate or by changing the degree to which aggregated CFTR is transported to aggresomes. To distinguish between these possibilities, first we investigated whether hook2 coexpression changed the biochemical properties of CFTR.

**Figure 4:**
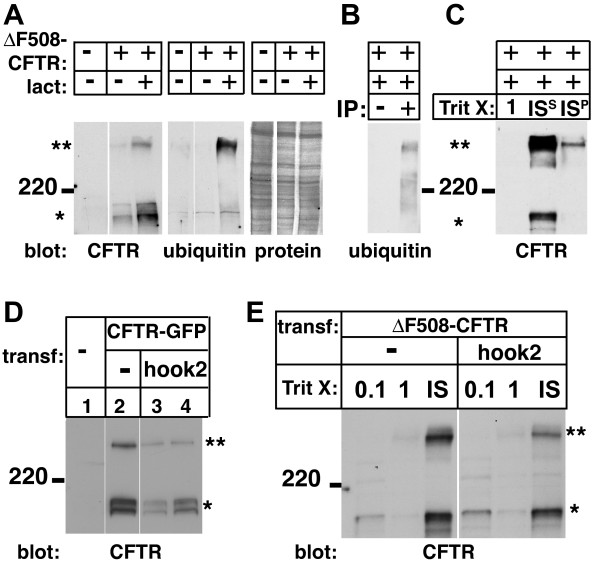
**Hook2 expression has no effect on the biochemical properties of CFTR**. (A) Vero cells transfected with ΔF508-CFTR were treated (+) or not (-) with lactacystin (lact), lysed and immunoblotted (blot:) for CFTR (left) and ubiquitin (middle). Stars indicate bands specifically seen in ΔF508-CFTR-expressing cells, compared to untransfected controls (-). As shown previously [39, 40] the upper band of CFTR runs at the same position as the ubiquitin-rich band in ΔF508-CFTR transfected and lactacystin treated cells. To control for loading membranes were stained with MemCode after immunoblotting (right). (B) Anti-ubiquitin antibodies also recognized the upper band of immunoprecipitated CFTR. (C) CFTR was expressed in the presence of lactacystin and analyzed by differential detergent extraction. Little CFTR was extracted by 1% Triton X-100 (1). Whereas the lower band of CFTR is soluble in 1% SDS (IS^S^), a fraction of the upper band is detected in the pellet of the 1% SDS extract (IS^P^) and needed to be solubilized in 6% SDS, as previously observed for other proteins in aggresomes [4, 14]. (D) CFTR western blot of whole cell homogenates of untransfected cells (lane 1) or cells expressing ΔF508-CFTR-GFP alone (lane 2) or together with hook2 (lanes 3 and 4 are duplicates) showed that hook2 had no effect on the migration of ΔF508-CFTR in SDS-PAGE gels. The apparent reduced level of CFTR-GFP after hook2 co-expression was not consistently observed. (E) Hook2 co-expression did not significantly change the fraction of ΔF508-CFTR detected in Western blots of the 0.1% or 1% Triton-X 100 soluble or insoluble (IS) fractions.

Lactacystin induced a shift to an insoluble high-molecular weight ubiquitinated form of ΔF508-CFTR [Figure 4A–B and [14,15]]. Extraction of cells also indicated that the accumulating high molecular form of CFTR was insoluble in 1% TritonX-100 and a small fraction was insoluble even in 1% SDS (Fig. 4C). Hook2 co-expression in the absence of lactacystin did not significantly change either the electrophoretic mobility pattern of CFTR (Fig. 4D) or its solubility (Fig. 4E), suggesting that effects of hook2 on CFTR distribution are more likely due to an alteration in the transport of CFTR aggregates to aggresomes.

**Figure 5:**
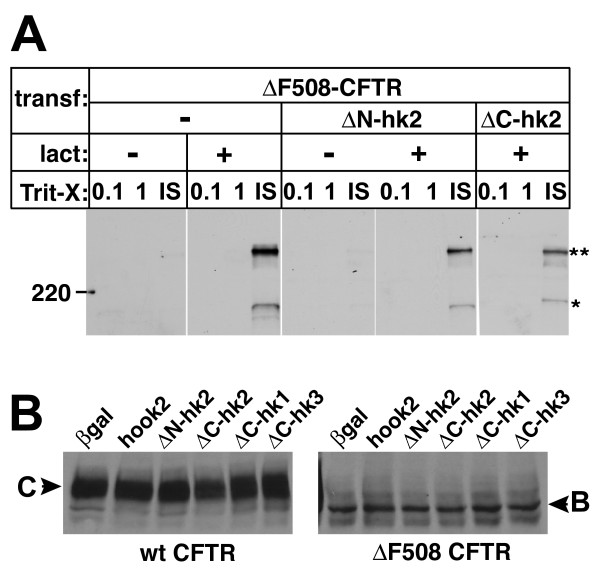
**Dominant-negative hook2 proteins do not alter the biochemical properties of CFTR**. (A) Western blots indicate that co-expression of hook2 truncations did not significantly change the ratio of CFTR in the high (**) to low-molecular mass (*) bands or the detergent solubility of ΔF508-CFTR. (B) Western blots of homogenates of cells co-transfected with CFTR and various hook proteins or controls showed that steady state levels of wt CFTR or ΔF508-CFTR were not altered by the indicated hook proteins. Band C is the mature CFTR protein and band B the core glycosylated form [41]. Equivalent loading was assured by evaluation of protein concentrations in cell lysates using the DC Protein Assay from BioRad. 20 μg total protein was loaded per well.

In addition, we also explored whether co-expression of hook2 truncations modified CFTR. Immunoblots of Triton X-100 extracts from cells co-expressing ΔF508-CFTR and either ΔN-hook2 or ΔC-hook2 showed that these dominant-negative constructs did not alter the electrophoretic mobility pattern of ΔF508-CFTR or its solubility, whether lactacystin was present or not (Fig. 5A). Furthermore, we speculated that changes in aggresome formation might be due to an inhibition of proteasome-mediated degradation of CFTR by over-expressed hook proteins. Therefore we compared levels of wild-type CFTR or ΔF508-CFTR proteins after co-transfection with hook2 or hook truncation mutants and various controls in the absence of lactacystin (Fig. 5B). Equivalent loading was assured by loading 20 μg total protein per lane. No change in steady-state CFTR protein levels was observed. These results indicated that the effect of hook2 truncation proteins on the subcellular distribution of ΔF508-CFTR was not due to changes in its biochemical properties or a change in proteasome activity.

## Discussion

Hook proteins constitute a family of coiled-coil proteins that have been implicated in the positioning of a variety of organelles [27]. In this study we present experiments suggesting that hook2 may function in the positioning or formation of aggresomes, pericentriolar accumulations of misfolded proteins, proteasomes and chaperones. Hook2 expression enhanced the recruitment of aggresome components to the centrosome, in addition to the molecular motor dynein which previously had been shown to accumulate in aggresomes and is necessary for their formation [4,16]. Furthermore, the accumulation of CFTR into aggresomes was inhibited by a hook2 C-terminal truncation that is modelled after a dominant-negative form of the *Drosophila *hook protein [23] and lacks the centriolin binding region of hook2 [21]. Expression of truncation mutants or full-length hook2 also altered the distribution of the centrosomal proteins PCM-1 and ninein [21], indicating that the effect of hook2 on aggresome formation may result from its primary role in regulating the activities of the centrosome.

A first clue to a possible function of hook2 in aggresome formation comes from its subcellular localization at the centrosome. Pericentrosomal accumulation in aggresomes has been observed for many overexpressed proteins [10], but several of our findings argue for hook2 playing a more active role than just being a misfolded substrate for aggresome formation. Importantly, centrosomal localization did not depend on overexpression-induced misfolding, but was also observed for endogenous hook2 [21]. In addition, dominant-negative hook2 proteins reduced the accumulation of co-expressed ΔF508-CFTR in aggresomes whereas wild-type hook2 enhanced it. This is unlikely to be due to the co-aggregation of misfolded hook2 and CFTR proteins, because aggregation is a specific process; even if two misfolded proteins are co-expressed they accumulate in separate aggregates [8].

A mechanism by which hook2 might enhance the formation of CFTR containing aggresomes is through the inhibition of proteasome activity by overexpressed hook2, as had been observed for some misfolded proteins [3,28]. However, for hook2, we do not consider this as a likely explanation for the following reasons: First, even when proteasome activity was inhibited by lactacystin, hook2 further enhanced aggresome formation compared to lactacystin on its own (Fig. 2), indicating that hook2 acts through a mechanism distinct from proteasome inhibition. Second, overexpressed C- and N-terminal truncations of hook2 which would be more likely to be misfolded than the wild-type protein and thus expected to promote aggresome formation, actually had the opposite effect and inhibited aggresome formation (Fig. 3). Third, whereas inhibition of proteasome activity resulted in a shift to a ubiquitinated high-molecular form and increased levels of CFTR [Fig. 4 and refs. [14,15]], no such changes were observed for ΔF508-CFTR after co-expression with wild-type or mutant forms of hook2 (Figs. 4, 5). Forth, if hook2 enhanced CFTR aggresomes by inhibiting the limited proteasome capacity in cells, then misfolded CFTR should have the reciprocal effect on hook2, which was not observed (Fig. 2). Together, these data argue against hook2 enhancing aggresome formation solely by acting as a misfolded substrate for aggresomes.

Instead, hook2 may act on the dynein-mediated retrograde transport of aggresome components. Aggresome formation depends on retrograde transport of misfolded protein aggregates and other aggresome components by cytoplasmic dynein, since nocadozole-induced depolymerization and dynamitin expression interfere with aggresome formation [4,16]. As a consequence of changes in microtubule dependent transport, dynein itself can accumulate in aggresomes [Fig. 1I, J and ref. [16]]. Thus, the inhibitory effect of truncated hook2 proteins on aggresome formation may, at least in part, reflect their disruptive effects on the radial array of microtubules [21].

Support for a model in which hook2 participates in the dynein-mediated retrograde transport of aggresome components comes from observations of its distant *C. elegans *homolog zyg-12. In early *C. elegans *embryos, zyg-12 is necessary for the positioning of centrosomes close to nuclei [24], a process that requires the recruitment of dynein and the dynein-associated proteins lis-1 and arp-1 to the nuclear membrane [24]. Two-hybrid analysis has suggested a direct interaction between zyg-12 and the dynein light intermediate subunit [24]. However, our attempts to demonstrate a direct interaction between hook2 and cytoplasmic dynein have not been successful and we did not notice significant co-localization of hook2 with peripheral CFTR aggregates. Alternatively, hook proteins may indirectly participate in retrograde transport by functioning as transient linker proteins or attachment factors during the loading of cargo onto the dynein/dynactin complex [18,27,29]. Such linker proteins may also contribute to the anchoring and stabilization of microtubules [29], functions traditionally attributed to centrosomes. Consistent with such a role of centrosomal hook2, dominant-negative hook2 constructs interfered with the regrowth of microtubules after their nocodazole-induced depolymerization [21]. In this context, it is also interesting to notice that the two hook2/3 chimeras that promoted aggresome formation in the absence of lactacystin (Fig. 3A) were previously found to localize to centrosomes [21].

A possible function of hook2 in modulating the retrograde transport of non-membranous protein particles is in line with observations that implicated other members of the hook family in modulating the movement and anchoring of organelles. Interference with hook3 function causes the dispersal of the Golgi complex [20], similar to the effect of overexpression of the dynamitin subunit of the dynein/dynactin complex [30,31]. Furthermore, hook1 is abundant in differentiating murine spermatids where it localizes to the ends of the microtubule manchette at the time of flagellar development. A deletion in the *hook1 *gene underlies the mouse mutation *abnormal spermatozoon head shape *(*azh*). *Azh *males have reduced fertility, a phenotype that correlates with malpositioning of the manchette, abnormal shaping of the spermatid nucleus, and fragile sperm tails [32]. The displacement of the manchette, a spermatid-specific microtubule array in *azh *mice [32] is consistent with an involvement of hook1 in the anchoring of microtubules.

These observations in *azh *mutant mice lacking hook1 and our finding that hook2 localizes to centrosomes, at least in part through a direct interaction with centriolin [21], suggest an alternative model in which the effects of hook proteins on transport are mediated through directly altering the functioning of the centrosome. One major role of centrosomes is to support the nucleation and organization of microtubules [33,34] and thus ultimately to control vectorial protein transport. In a previous study, we could not detect an effect of hook2 on microtubule nucleation but our data revealed a contribution of hook2 to the maintenance of the radial arrangement of microtubules [21]. However, since cytoplasmic dynein also transports centrosomal proteins and microtubules [35-37], it is not straightforward to distinguish direct effects on centrosomes from the indirect consequences of abnormal retrograde transport. Future studies to unravel the molecular mechanisms by which hook2 contributes to the formation of aggresomes should take into consideration that hook2 may modulate microtubule-dependent retrograde transport.

## Conclusion

Taken together with previous findings that hook2 localizes to centrosomes, binds directly to centriolin and that interference with hook2 function disturbs the radial organization of microtubules, our results indicate that centrosomally localized hook2 promotes the generation or maintenance of aggresomes when misfolded proteins accumulate in cells. Based on the functions observed for other hook homologs [20,24,32] and the defects in the radial organization of microtubules upon interference with hook2 function [21], we propose that hook2 function is required for the microtubule-based delivery of protein aggregates to pericentriolar aggresomes.

## Methods

### Cell lines

The following cell lines were used in this study, with tissue and species source for each line in parenthesis: HEK 293 (human embryonic kidney), VERO (adult African green monkey kidney); these cell lines were obtained from ATCC (American Type Culture Collection; Manassas, VA) and maintained as recommended by ATCC.

### Sources of Antibodies

Polyclonal antibodies against hook2 (aa 427–719) and hook3 (423–630) have been described [20]; these were diluted 1:200–1:400 to detect endogenous hooks and 1:400–1:600 to detect overexpressed hooks by immunohistochemistry and 1:2,000–1:10,000 for immunoblotting. Anti-tubulin, dynein 74.1, anti-heat shock protein 70 and rabbit ubiquitin antibodies were from Sigma (Saint Louis, MO; sigma-aldrich.com); GM130 from BD Transduction Labs (bdbiosciences.com); FK1, anti-ubiquitin, and anti-20S proteasome antibodies from Biomol International (biomol.com); mouse anti-CFTR (mAb3A7) or mouse anti-human CFTR (C-24-1) at 1:150 for immunostaining and immunoprecipitation, 1:1000 for immunoblotting (Research Diagnostics, Inc.; Flers, NJ). Secondary antibodies for immunoblots were horseradish peroxidase labeled goat anti-mouse and anti-rabbit from Bio-Rad, each used at a dilution of 1:10,000. Secondary antibodies for immunofluorescence were Alexa-488, -564, or -594 conjugated goat anti-mouse or goat anti-rabbit antibodies from Molecular Probes (Eugene, OR), each used at a dilution of 1:400.

### Immunoblotting and immunoprecipitation

Equal amounts of cell extracts, or cell homogenates were separated by SDS-PAGE gels (Criterion Precast Gels; Bio-Rad; Hercules, CA) and transferred to nitrocellulose (Schleicher and Schuell; Keene, NH). Prior to the application of antibodies, membranes were blocked in 3% non-fat dry milk in wash buffer (20 mM Tris-HCl pH 7.5; 150 mM NaCl; 1% Tween-20) for 1 h at RT or over-night at 4°C. Primary antibodies were applied in block for 1–2 hrs at RT. Secondary antibodies (goat anti-mouse or anti-rabbit IgG-HRP conjugate (Bio-Rad) were diluted 1:10,000 and applied for 1 hour at RT. Blots were rinsed at least three times in wash buffer. Immunoreactive bands were visualized on Blue Biofilm (Denville Scientific; Metuchen, NJ) after the application of SuperSignal West Pico Stable or West Femto Stable ECL substrate (Pierce; Rockford, IL). Finally, the efficiency of protein transfer to blots was assessed by staining membranes with the MemCode protein stain (Pierce; Rockford, IL). Immunoprecipitation of CFTR followed the protocol described by Ward et al. (1995).

### cDNA constructs and transfections

Constructs expressing various hook2 and hook3 wild-type or mutant proteins have been described [20,21]. In brief, the constructs 223, 322, 332, 233, 323, and 232 are chimeras of hook2 and hook3: 223 contains hook2 aa 1–567 and hook3 aa 573–719; 322 contains hook3 aa 1–213 and hook2 aa 210–719; 332 contains hook3 aa 1–572 and hook3 aa 567–719; 233 contains hook2 aa 1–210 and hook3 aa 215–719; 323 is hook3 with a hook2 insert from aa 213 to 572; 232 is hook2 with a hook3 insert from aa 210 to 567. Chimeras were designed to approximate locations of the previously mapped N-terminal microtubule binding region, the central coiled-coil homodimerization domain, and the proposed organelle binding C-terminal domain boundaries [20-22]. ΔC-hook2, ΔN-hook2 and ΔCl-hook2 are deletion constructs of hook2: from ΔC-hook2 aa 533–719 were deleted, from ΔN-hook2 aa 1–161, and from ΔCl-hook2 aa 439–554, including 507–548 that comprises the C-terminal region of the coil-coil domain. Each of the constructs was inserted into mammalian expression vector pcDNA3.1 that contains a CMV promoter (Invitrogen, Carlsbad, CA; invitrogen.com). Plasmids pCMVNot6.2 and pCMVNot6.2-ΔF508 containing expressible human CFTR cDNAs were the generous gift of Dr. Johanna Rommens (The Hospital For Sick Children, Toronto). A construct expressing a GFP-tagged CFTR [38] was a gift of Bruce Stanton (Dartmouth Medical School).

Vero and HEK cells were transfected with Fugene (Roche; Indianapolis, IN) using a ratio of 1 μg DNA per 3 μl Fugene for HEK cells and a 1:6 ratio for Vero cells.

### Differential detergent extraction of live cells and preparation of whole cell lysates

Vero cells grown on 35–60 mm plates were placed on a slide warmer and rinsed several times with 37°C PHEM buffer (60 mM PIPES, 25 mM HEPES, 10 mM EGTA, and 1 mM MgCl_2_, pH 7.4). Cells were sequentially extracted for 3 min with increasing concentrations of detergents (0.1% and 1% Triton X-100 and finally 1% SDS) in 100–300 μl PHEM buffer containing protease inhibitors for mammalian cells (Roche; Indianapolis, IN). Each fraction was centrifuged at 20,000 × g and supernatants of each fraction and in some cases the pellet of the 1% SDS fraction were further solubilized in loading buffer with 6% SDS prior to loading. Whole-cell lysates were obtained by scraping cells directly into loading buffer after washes in serum-free medium at 37°C.

### Immunocytochemistry and microscopy

For immunohistochemistry, cells grown on 4-well Lab-Tek II chamber slides (Nalgene-Nunc) up to a density of ~5000 cells/cm^2 ^were rinsed with PHEM buffer at 37°C, then fixed in methanol at -20°C for 8 min. After two rinses with PBS, formaldehyde-fixed cells were permeabilized in 0.2% Triton X-100 in PBS for 10 min at room temperature. Nonspecific binding was blocked with 2.5% gelatin/3% BSA/0.2 Triton X-100 in PBS pH 7.4 for at least 45 min at 37°C. Antibodies were diluted in blocking solution; primary antibodies were applied overnight at 4°C and secondary antibodies for 1 hr at room temperature. Washes were in 0.2% Tween-20 in PBS. Nuclei were stained with 0.5 μg/ml Hoechst (Aldrich Chemicals, St. Louis, MO). After rinses in 50 mM Tris (pH 8.0), sections were mounted with Gel/Mount (Biomedia, Foster City, CA).

Fluorescent cell preparations were viewed through 20× (NA 0.5), 40× (NA 1.0) or 100× (NA. 1.3) PL/Fluotar Leica objectives on a Leica Leitz, DMR microscope (Thornwood, NY). Images were captured with an AxioCam camera (Zeiss) controlled by Axiovision 3.0. All images used for determining the co-localization of proteins were captured in sequential mode with a Leica CS SP2 confocal scanner on a Leica DMIRE2 microscope through a 63× HCX PL APO (NA1.32) objective. Images were assembled into panels in Adobe Photoshop (Adobe Systems, Mountain View, CA) and all images within a panel were adjusted together for contrast and brightness.

## Authors' contributions

GS performed the majority of the biochemical and cell analytical experiments with the help of MY and BH. GS organized and analyzed data, and drafted the initial manuscript. AD performed the initial experiments revealing an effect of hook2 on CFTR. HK designed and constructed plasmids and MY prepared DNA. WCW performed experiment shown in figure 6B. GS, HK and PT designed experiments. GS and HK finalized the manuscript and discussed it with the other authors.
